# Current Status and Management Strategies of Obstetric Hemorrhage Using Contrast-enhanced Dynamic Computed Tomography in a Representative Tertiary Perinatal Medical Center in Japan

**DOI:** 10.31662/jmaj.2024-0114

**Published:** 2024-12-06

**Authors:** Naohiro Suzuki, Yoshitsugu Chigusa, Haruta Mogami, Maya Komatsu, Masahito Takakura, Ken Shinozuka, Shigeru Ohtsuru, Masaki Mandai, Eiji Kondoh

**Affiliations:** 1 Department of Gynecology and Obstetrics, Graduate School of Medicine, Kyoto University, Kyoto, Japan; 2 Department of Primary Care and Emergency Medicine, Graduate School of Medicine, Kyoto University, Kyoto, Japan; 3 Department of Obstetrics and Gynecology, Faculty of Life Sciences, Kumamoto University, Kumamoto, Japan

**Keywords:** atonic bleeding, balloon tamponade, contrast-enhanced dynamic computed tomography, obstetric hemorrhage, transcatheter arterial embolization

## Abstract

**Introduction:**

Obstetric hemorrhage is a leading cause of pregnancy-related mortality. Our hospital protocol states that patients with obstetric hemorrhage undergo initial imaging with contrast-enhanced dynamic computed tomography (CE-dCT) to ascertain the presence and location of active bleeding, followed by tailored therapeutic interventions. Herein, we aimed to elucidate the prevailing status and clinical outcomes of obstetric hemorrhage cases at our institution, which are characterized by a distinctive, methodical treatment approach.

**Methods:**

This retrospective observational study included 150 patients with obstetric hemorrhage. Clinical information, including bleeding volume, hemorrhage etiology, therapeutic intervention, transfusion quantity, patient outcome, and CE-dCT findings, were explored.

**Results:**

The leading cause of obstetric hemorrhage was atonic bleeding (55%), followed by vaginal hematoma (13%) and retained placenta (11%). The median amount of bleeding was 2,803 mL, and the median volume of red blood cells (RBC) and fresh frozen plasma (FFP) required was 6 units. Blood loss and transfusion volume were similar regardless of the cause of obstetric hemorrhage. Conservative management, such as uterotonics or balloon tamponade, achieved hemostasis in 57% of patients, whereas 43% required invasive interventions, such as transcatheter arterial embolization. CE-dCT was performed on 85% of patients, and extravasation was detected in 53%. Moreover, “PRACE,” characterized by Postpartum hemorrhage, Resistance to treatment, and Arterial Contrast Extravasation on CE-dCT scans, potentially requires massive blood transfusions and invasive treatment.

**Conclusions:**

Although obstetric hemorrhage encompasses a diverse array of pathologies, medical practitioners must recognize that approximately 3,000 mL of blood is lost and at least 6 units of RBC and FFP are required, irrespective of the cause. CE-dCT plays a pivotal role in elucidating the etiology of obstetric hemorrhage and guiding therapeutic interventions.

## Introduction

“Obstetrics is a bloody business” is a well-known maxim by Dr. Jack Pritchard in Williams Obstetrics in 1976, which is one of the most classic and authoritative obstetrics textbooks ^(1)^. This quote indicates that hemorrhage has long been the leading cause of pregnancy-related deaths. Globally, obstetric hemorrhage accounts for 27% of all maternal deaths, although there are marked differences in maternal mortality between countries and regions ^(2), (3)^. In Japan, there were 583 maternal deaths in 2010-2022, of which 558 women were analyzed and obstetric hemorrhage was reported for 18% of causes ^(4)^. Thus, obstetricians must focus on obstetric hemorrhage to mitigate maternal mortality rates.

Obstetric hemorrhage, also known as postpartum hemorrhage, is generally defined as ≥500 mL bleeding within 24 h after delivery ^(5)^. However, severe hemorrhages of ≥1,000 mL are clinically problematic. Obstetric hemorrhage is a common term for bleeding that occurs during pregnancy and after childbirth; therefore, it includes various pathological conditions, such as uterine atony, vaginal lacerations, puerperal genital hematomas, placenta accreta spectrum, retained placenta, retained products of conception, coagulopathy resulting from amniotic fluid embolism, and placental abruption ^(6)^. These leading causes of obstetric hemorrhage are classified into the following four Ts: tone (uterine atony); trauma (laceration, hematoma, rupture); tissue (retained placenta, abnormally adherent placenta); and thrombin (defects of coagulation) ^(7), (8)^.

Although obstetric hemorrhage can be life-threatening, most obstetric hemorrhage-related maternal mortality and severe morbidity can be prevented if treated timely and effectively ^(9)^. Fundamental strategies have been widely advocated and implemented for effective obstetric hemorrhage management: prompt medical personnel mobilization and patient transfer to a tertiary care facility; appropriate blood transfusion support; intrauterine balloon tamponade; and possible surgical interventions, including hysterectomy, without delay ^(6), (8)^. Nevertheless, obstetric hemorrhage encompasses various pathological conditions; additionally, identifying the cause of bleeding is important.

We previously reported that contrast-enhanced dynamic computed tomography (CE-dCT) can effectively identify the etiology of obstetric hemorrhage ^(10)^. Beyond obstetric hemorrhage, CE-dCT has diagnostic capabilities in various areas, such as abdominopelvic trauma ^(11)^, liver injury ^(12)^, and gastrointestinal bleeding ^(13), (14)^. Ultrasound is the primary imaging modality in obstetrics, considering it is safe, noninvasive, and inexpensive to perform. However, identifying active hemorrhage through ultrasound examination is challenging because of the technique-dependent nature of ultrasound and limited scanning area. Therefore, at our institution, patients with obstetric hemorrhage undergo initial imaging with CE-dCT to ascertain the presence and location of active bleeding, followed by tailored therapeutic interventions ^(10), (15)^, which is a distinctive feature of our hospital. Therefore, this study elucidated the prevailing status and clinical outcomes of obstetric hemorrhage cases at our institution, characterized by a distinctive, methodical treatment approach.

## Materials and Methods

### Study design and patients

The Ethics Committee of Kyoto University approved this retrospective observational study (R3996). Informed consent was obtained through the opt-out form on the website. Pregnant patients or patients within 6 weeks after the abortion or birth process―regardless of route of delivery―and patients who were transferred to Kyoto University Hospital from primary or secondary medical facilities between August 2013 and June 2022 with uterine, vaginal, intra-abdominal, or retroperitoneal bleeding resulting from obstetric causes―regardless of the amount of bleeding―were included in the study. However, patients with ectopic pregnancies or significant missing clinical data were excluded. We retrospectively reviewed the data of all eligible patients and extracted and analyzed relevant clinical information from patient medical charts, including gestational age at the onset of obstetric hemorrhage, causes of bleeding, therapeutic intervention, amount of bleeding, and transfusion volume.

### Evaluation of computed tomography

Previously, we advocated the intractable subtype of postpartum uterine bleeding named “PRACE,” which is characterized by Postpartum hemorrhage, Resistance to treatment, and Arterial Contrast Extravasation on dynamic CT scans ^(15)^. Two obstetricians, NS and YC, evaluated all CT images of eligible patients to detect PRACE. Herein, we defined PRACE as postpartum uterine bleeding in the presence of contrast extravasation, which refers to extravascular leakage of contrast medium, suggesting active bleeding, on CT in the early phase (40 s) and increasing spread of extravasation in the late phase (90 s) ^(15)^. We divided the uterus into two sections along the long axis (upper and lower) and evaluated the bleeding points ^(15)^. Supplementary Figure 1 shows the representative CT images of patients diagnosed with PRACE. Active extravasation of the contrast medium was detected in the early phase (Supplementary Figure 1A and Supplementary Figure 1C) and was widespread in the late phase (Supplementary Figure 1B and Supplementary Figure 1D).

### Statistical analyses

Data are presented as median [range]. Continuous and categorical variables were assessed using the Mann-Whitney and Fisher’s exact tests between the PRACE (+) and PRACE (−) groups in vaginal delivery using GraphPad Prism (version 9, San Diego, CA, USA). A P-value of <0.05 was considered statistically significant.

## Results

### Patients and standard protocol for obstetric hemorrhage management

Between August 2013 and June 2022, 997 patients were transferred to our hospital for various obstetric emergencies, such as preterm labor, preeclampsia, and exacerbations of maternal complications. Of these, 187 cases met the inclusion criteria, and the remaining 810 cases were obstetric emergencies other than obstetric hemorrhage. In addition, 37 patients were excluded because of ectopic pregnancy (n = 32) or significant missing clinical data (n = 5). Finally, 150 patients with obstetric hemorrhage were included. The standard protocol for managing obstetric hemorrhage at our institution is as follows: patients with obstetric hemorrhage undergo initial CE-dCT to determine the presence and location of active bleeding. Nevertheless, if hemostasis occurs before the patient’s arrival at our hospital or if hemodynamic instability necessitates immediate intervention due to severe conditions such as uterine rupture or intra-abdominal hemorrhage, CE-dCT may not be feasible.

When CE-dCT results indicate that hemorrhage source is external to the uterus, such as a vaginal hematoma, surgery is first treatment of choice regardless of the presence or absence of extravasation. For uterine bleeding without extravasation, conservative treatment with uterine contractile agents is indicated. In patients with uterine bleeding and extravasation in the lower half of the uterus, balloon tamponade is the first choice. However, when extravasation occurs in the upper half of the uterus or in cases of PRACE, transcatheter arterial embolization (TAE) should be the preferred intervention instead of balloon tamponade.

### Patient backgrounds

Table 1 presents the patient characteristics. The median age was 35.3 [22-45] years and 100 (67%) patients were primiparas. Regarding delivery mode, 90 (60%) and 56 (37%) patients underwent vaginal delivery and cesarean section, respectively, 3 underwent induced abortion in the second trimester, and 1 underwent dilatation and curettage in the first trimester. Very few patients had pregnancies of <37 weeks, and 142 (95%) were full-term. The median amount of bleeding was 2,803 [605-29,015] mL, and the median transfusion volumes of red blood cells (RBC) and fresh frozen plasma (FFP) required for treatment were 6 [0-108] and 6 [0-106] units, respectively. One maternal death occurred because of amniotic fluid embolism and subsequent disseminated intravascular coagulation. Three patients underwent hysterectomy: two patients with placenta accreta spectrum (PAS) and placenta previa and one with heterotopic cervical pregnancy.

**Table 1.** Patient Characteristics.

Variable	n = 150
Age	35.3 [22-45]
Primipara (n)	100
Multipara (n)	50
Vaginal delivery (n)	90
Cesarean section (n)	56
Gestational age
-12w (n)	2
12-22w (n)	2
22-37w (n)	4
37w- (n)	142
Amount of bleeding (mL)	2,803 [605-29,015]
Blood products
RBC (unit)	6 [0-108]
FFP (unit)*	6 [0-106]
PC (unit)	0 [0-125]
cryoprecipitate (bag)	0 [0-8]

Data are presented as median [range]. RBC: red blood cells, FFP: fresh frozen plasma, PC: platelet concentrate * One bag of cryoprecipitate (50 mL) was converted to four units of FFP.

### Causes and treatment of obstetric hemorrhage

**Figure 1.**
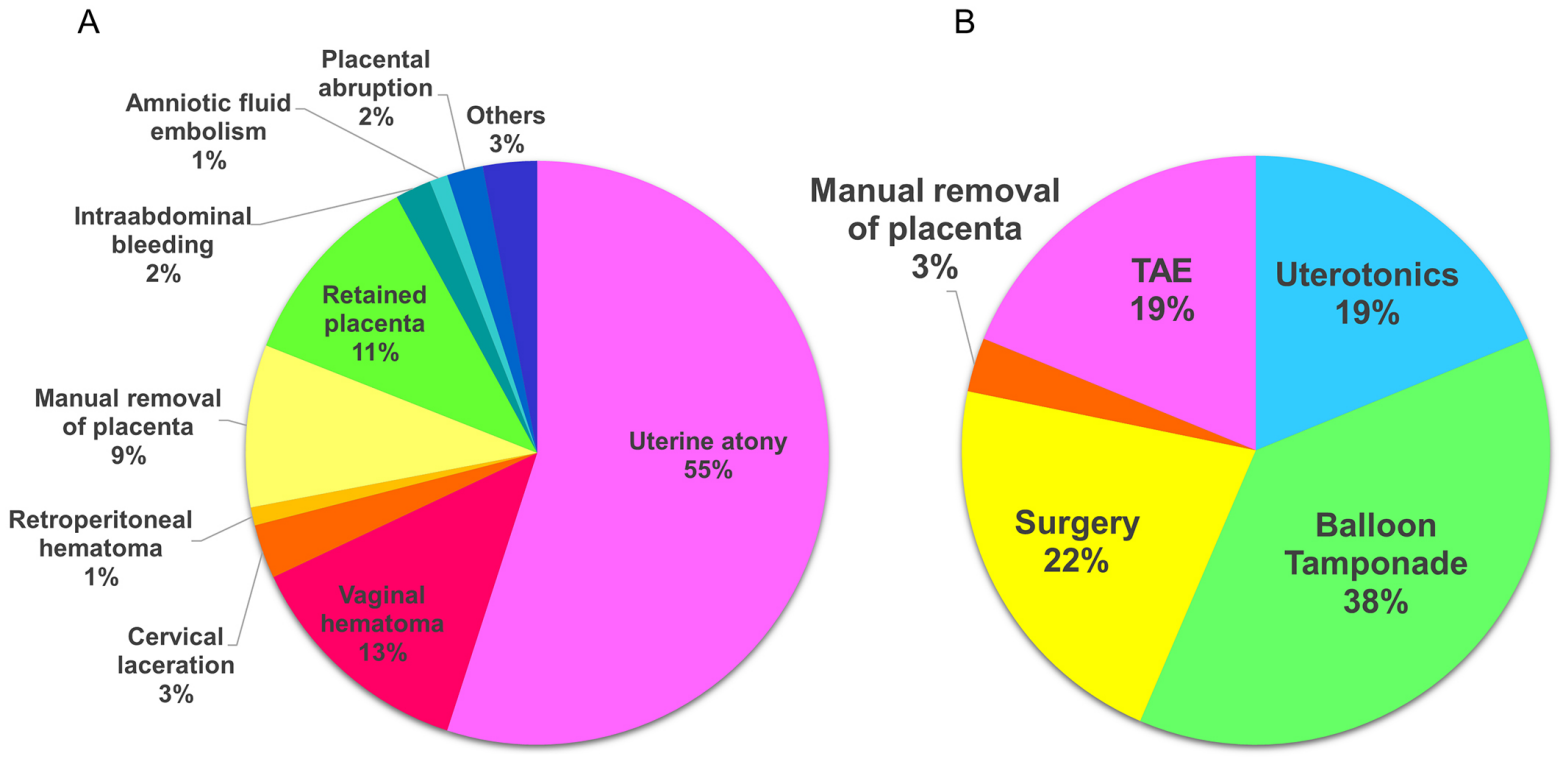
Causes of obstetric hemorrhage (A) and treatment types (B) TAE: transcatheter arterial embolization.

Figure 1A depicts the breakdown of the causes of bleeding, with atonic bleeding being the most common cause of obstetric hemorrhage (55%), which is defined as abnormal uterine bleeding caused by failure of uterine contraction after childbirth, followed by vaginal hematoma (13%). In addition, there was a high incidence of hemorrhage related to placental remnants or adherent placenta: retained placenta (11%) and bleeding when the retained placenta was manually removed (9%).

Table 2 shows these causes categorized into four Ts: tone accounted for 55%, trauma and tissue for approximately 20% each, and thrombin for the remaining 3%. Among the four Ts, thrombin had the highest blood loss and transfusion volume, implying that thrombin is the intractable pathology in obstetric hemorrhage.

**Table 2.** Causes of Obstetric Hemorrhage Based on four Ts.

4Ts	n (%)	Amount of bleeding (mL)	RBC (unit)	FFP (unit)
Tone	83 (55.3)			
Atonic uterine bleeding (n = 83)		2,751 [605-8,013]	6 [0-24]	6 [0-26]
Trauma	33 (22.0)			
Puerperal genital hematomas (n = 21)				
Cervical laceration (n = 4)				
Intra-abdominal hemorrhage (n = 3)		2,655 [800-29,015]	8 [0-108]	6 [0-106]
Retroperitoneal hematomas (n = 2)				
Uterine rupture (n = 2)				
Heterotopic cervical pregnancy (n = 1)				
Tissue	29 (19.3)			
Retained placenta (n = 16)		3,000 [1,500-5,927]	6 [0-22]	6 [0-28]
Manual removal of placenta (n = 13)				
Thrombin	5 (3.3)			
Placental abruption and subsequent DIC (n = 3)		3,300 [2,412-9,908]	12 [0-80]	18 [0-56]
Amniotic fluid embolism and subsequent DIC (n = 2)	

Data are presented as median [range]. RBC: red blood cells, FFP: fresh frozen plasma, DIC: disseminated intravascular coagulation

Figure 1B depicts the treatments employed. In 38% of patients, balloon tamponade effectively achieved hemostasis. Uterine contractile agents alone were successful in 19% of patients. In 57% of patients, hemostasis was achieved with conservative treatment. In 22% of patients, surgery was required, including removal of hematoma and hemostasis in the vagina, repair of cervical laceration, and hysterectomy. TAE was performed in 19% of patients.

### CE-dCT findings and corresponding treatment and outcome

**Figure 2.**
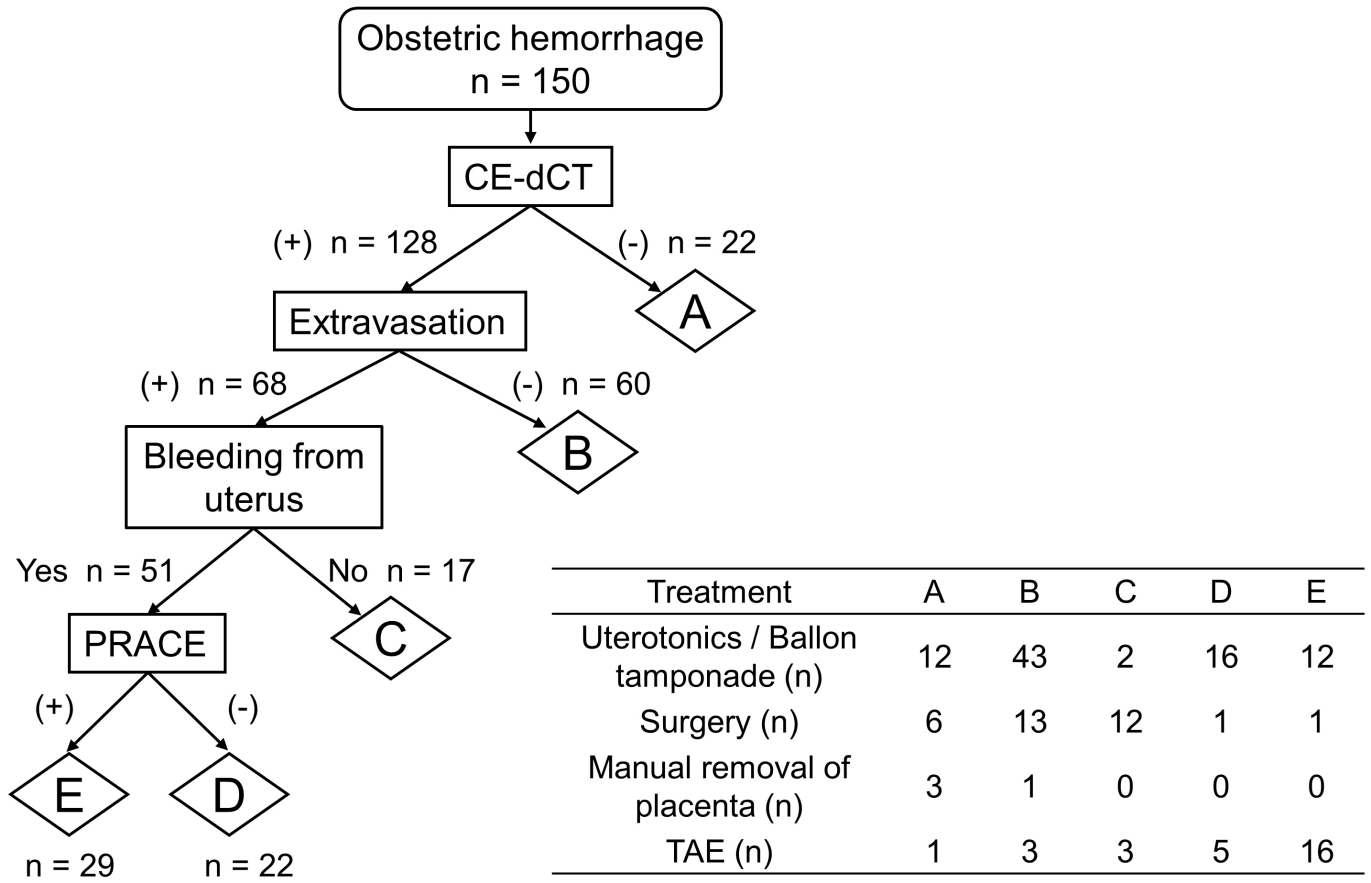
Patient flow chart based on CE-dCT and treatment types CE-dCT: contrast-enhanced dynamic computed tomography, PRACE: Postpartum hemorrhage, Resistance to treatment, and Arterial Contrast Extravasation on dynamic computed tomography scans, TAE: transcatheter arterial embolization.

We examined the differences in the presentation and management of patients with obstetric hemorrhage based on the presence or absence of PRACE. Figure 2 shows patient flow per CE-dCT. Of 150 patients, 128 (85%) underwent CE-dCT. Among them, 68 (53%) had extravasation: 51 and 17 from uterine bleeding and bleeding from other sides, respectively. Of 51 patients with uterine extravasation, 29 met the PRACE criteria. The bleeding site and amount did not significantly differ between two groups (Table 3). However, there was a significant difference in treatment methods between the two groups (*P* = 0.046); 17 (53%) patients received invasive treatment such as TAE or surgery in PRACE (+) group, whereas 13 (68%) received conservative treatment in PRACE (−) group (Table 3). Moreover, the RBC and FFP required for treatment were significantly high in the PRACE (+) group (*P* = 0.027 and 0.0091; Table 3).

**Table 3.** Obstetric Hemorrhage Differences with or without PRACE.

	PRACE (+)	PRACE (−)	*p*-value
	n = 29	n = 22	
Bleeding site			
Upper	24	17	0.73
Lower	5	5	
Types of treatment			
TAE/hysterectomy	17	6	0.046
Uterotonics/balloon tamponade	12	16	
Amount of bleeding (mL)	3,444 [960-29,015]	3,000 [605-9,908]	0.40
Transfusion volume			
RBC (unit)	12 [0-108]	4 [0-22]	0.027
FFP (unit)	10 [0-106]	5 [2-28]	0.0091

Data are presented as median [range]. PRACE: Postpartum hemorrhage, Resistance to treatment, and Arterial Contrast Extravasation on dynamic computed tomography scans, TAE: transcatheter arterial embolization, RBC: red blood cells, FFP: fresh frozen plasma

## Discussion

This study investigated the conditions of patients with obstetric hemorrhage treated at a single tertiary medical center with a distinctive management approach using CE-dCT. The findings indicated that the leading cause of obstetric hemorrhage was uterine atony, and the median amount of bleeding was 2,803 mL. Conservative treatment was successful in 57% of patients, and the median volume of RBC and FFP required for treatment was 6 units each. Furthermore, among obstetric uterine hemorrhages, PRACE potentially requires massive blood transfusions and invasive treatments, including TAE.

Herein, among the four Ts classifications of causes of bleeding, tone was the predominant cause, representing 55% of obstetric hemorrhage, whereas tissue accounted for approximately 20%. Previously, the breakdown of the four Ts was 70% tone, followed by 20% trauma, 10% tissue, and 1% thrombin ^(8)^. However, our data indicate that the tone rate declined and the tissue rate nearly doubled. Furthermore, in our recent study on the epidemiological dynamics of obstetric hemorrhage in Japan using a national database of health insurance claims, the percentage of obstetric hemorrhage because of tissue, such as PAS, and retained placenta significantly increased ^(16)^. This shift is partially attributable to the spread of assisted reproductive technologies (ART) and the accompanying increase in PAS. The incidence of obstetric hemorrhage caused by tissue will potentially increase in Japan owing to an increase in placenta previa associated with high maternal age.

In 57% of patients with obstetric hemorrhage, conservative treatment, such as uterine contractions, balloon tamponade, or both, was successful. The dissemination of maternal life-saving simulation education by the Japan Society of Obstetrics and Gynecology and the Japan Association of Obstetricians and Gynecologists throughout the country since 2012 may have played a role in this trend ^(16)^. However, no study has comprehensively investigated the distribution of treatment modalities for obstetric hemorrhage in Japan. Conservative management is predominantly effective for obstetric hemorrhage caused by tone and tissue. In such cases, CT-dCT plays a pivotal role in determining treatment strategies. If there is no extravasation of the uterus, conservative treatment can achieve hemostasis. For atonic bleeding, there is usually only one bleeding point identified in the uterus by contrast extravasation ^(10), (15)^. In particular, if bleeding occurs in the lower uterus, balloon tamponade is a potentially effective first treatment option. PRACE, distinguished by contrast extravasation on CE-dCT in the early phase and its subsequent increasing spread in the late phase, should be treated with proactive consideration of the potential requirement for TAE. Nevertheless, TAE has potential complications, such as uterine necrosis, infection, and a high rate of PAS in subsequent pregnancies ^(17)^. Hence, appropriately selecting cases in which TAE is unavoidable via CE-dCT is imperative.

To our knowledge, our study is the first to determine the approximate volume of blood loss and corresponding transfusion requirements concerning obstetric hemorrhage. Accurately determining the amount of blood loss in unexpected massive hemorrhages is difficult. Visual estimation is subjective and imprecise and often underestimates the amount of blood loss. Furthermore, the amount of hemorrhage forming a large hematoma cannot be ascertained. However, inaccurate blood loss estimates delays the response to obstetric hemorrhage, which should be prompt ^(18)^. Moreover, our data, which showed a median blood loss of 2,803 mL, may be related to the inaccuracy of bleeding volume measurement. Nevertheless, our data are remarkable and serve as a reminder to healthcare providers, stating that for obstetric hemorrhage, at least 3,000 mL of blood is lost. Furthermore, these cases required 6 units each of RBC and FFP. Blood loss and transfusion volumes were similar regardless of the cause, tone, trauma, or tissue, except for thrombin. In our hospital, we empirically prepare 6 units of noncross-matched RBC and FFP upon admitting a patient with obstetric hemorrhage. This study confirms the adequacy of our protocol.

This study has a few limitations. First is the potential case bias attributable to its tertiary, single-center, and retrospective design. In actual clinical practice, patients with obstetric hemorrhage can require intensive care management without transfer to a tertiary care facility. Second, given that all patients included in this study were transported from other medical facilities, sufficient detailed clinical information was not collected, such as the presence or absence of ART, neonatal condition, and complications during pregnancy. Therefore, analyzing the risk factors for obstetric hemorrhage was impossible. Surpassing the limitation of our research will require augmenting the number of facilities using CE-dCT to manage obstetric hemorrhage, which is what we have done, and organizing multicenter trials to collect further data. A prospective study design and comprehensive patient information from primary medical facilities could provide detailed insight into the reality of obstetric hemorrhage and help identify risk factors associated with this condition.

In conclusion, as epitomized by the four Ts, obstetric hemorrhage encompasses a diverse array of pathologies, and prompt treatment is crucial based on the unique characteristics of each. Conservative management demonstrated effectiveness in approximately 57% of patients; conversely, 43% required invasive interventions such as TAE or surgery. A timely transition to invasive treatment based on CE-dCT findings is paramount. Moreover, a minimum reserve of 6 units each of RBC and FFP should be readily available.

## Article Information

### Conflicts of Interest

None

### Acknowledgement

We thank Editage (www.editage.com) for English language editing.

### Author Contributions

All authors contributed to the conception and design of the study. Naohiro Suzuki and Yoshitsugu Chigusa collected and interpreted the data and wrote the first draft of the manuscript. Ken Shinozuka and Shigeru Ohtsuru carried out a professional analysis of the data from the perspective of acute care physicians. Haruta Mogami, Masahito Takakura, and Maya Komatsu made an essential contribution to the generation of figures and revision of the manuscript. Masaki Mandai and Eiji Kondoh supervised the entire process. Naohiro Suzuki and Yoshitsugu Chigusa contributed equally to this work.

### Approval by Institutional Review Board (IRB)

All procedures complied with the ethical standards of the responsible committees on human experimentation (institutional as well as national) and the 1964 Declaration of Helsinki and its later amendments. Informed consent was obtained through the opt-out form on the website. The Ethics Committee of Kyoto University approved this study (R3996).

## Supplement

Supplementary Figure 1. Representative images of contrast-enhanced dynamic computed tomography (CD-dCT) in a patient with obstetric uterine hemorrhages and PRACE; active extravasation of contrast was detected in the early phase (Arrows; Supplementary Figure 1A and Supplementary Figure 1C) and was widespread in the late phase (Arrowheads; Supplementary Figure 1B and Supplementary Figure 1D).
PRACE: Postpartum hemorrhage, Resistance to treatment, and Arterial Contrast Extravasation on dynamic computed tomography scans.
