# Supplementary figures and images for: Current Status and Management Strategies of Obstetric Hemorrhage Using Contrast-enhanced Dynamic Computed Tomography in a Representative Tertiary Perinatal Medical Center in Japan
Source: JMA J. 2024 Dec 6;8(1):242–8. doi: 10.31662/jmaj.2024-0114 (PMC11799725; doi:10.31662/jmaj.2024-0114)

Supplementary Figure 1.

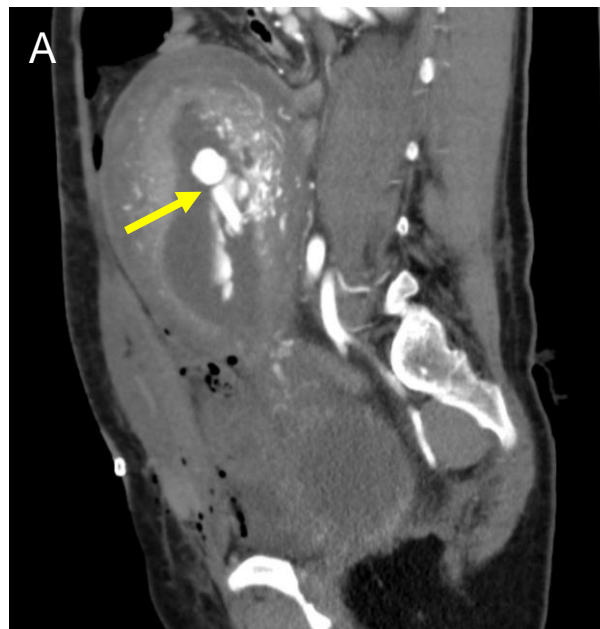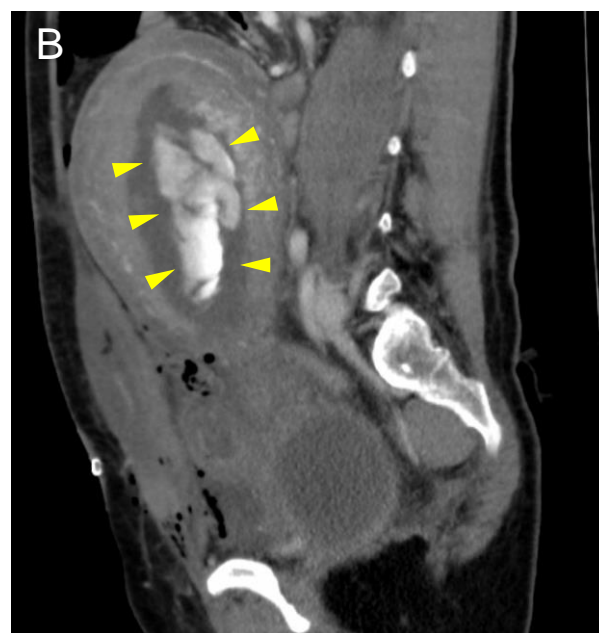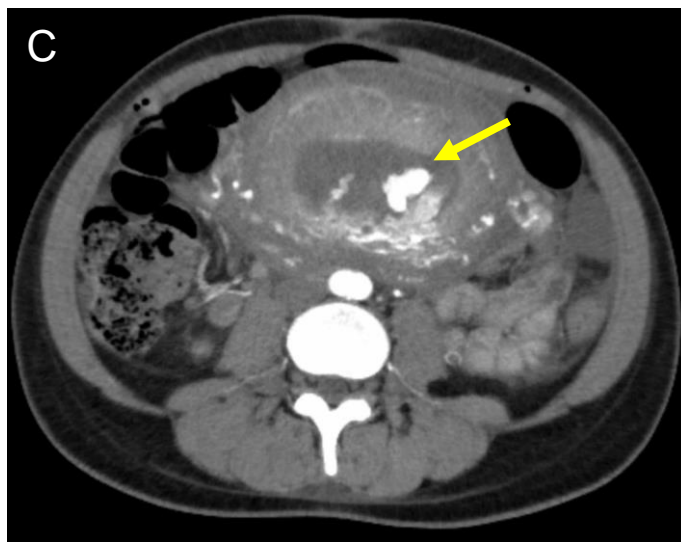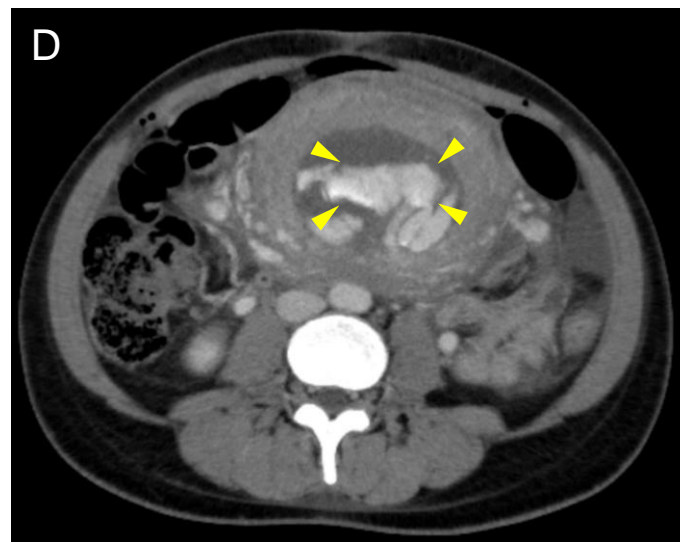

Supplement: Supplementary Figure 1. — Representative images of contrast-enhanced dynamic computed tomography (CD-dCT) in a patient with obstetric uterine hemorrhages and PRACE; active extravasation of contrast was detected in the early phase (Arrows; Supplementary Figure 1A and Supplementary Figure 1C) and was widespread in the late phase (Arrowheads; Supplementary Figure 1B and Supplementary Figure 1D). PRACE: Postpartum hemorrhage, Resistance to treatment, and Arterial Contrast Extravasation on dynamic computed tomography scans. [file 2433-3298-8-1-0242-s001.pdf]
